# Investigating the Impact of COVID-19 Lockdown on the Psychological Health of University Students and Their Attitudes Toward Mobile Mental Health Solutions: Two-Part Questionnaire Study

**DOI:** 10.2196/19876

**Published:** 2020-10-20

**Authors:** Nidal Drissi, Ayat Alhmoudi, Hana Al Nuaimi, Mahra Alkhyeli, Shaikha Alsalami, Sofia Ouhbi

**Affiliations:** 1 United Arab Emirates University Al Ain United Arab Emirates

**Keywords:** COVID-19, GHQ-12, mobile, apps, m-health, m-mental health, UAE, attitudes, university students, questionnaire

## Abstract

**Background:**

The COVID-19 outbreak was first reported to the World Health Organization on December 31, 2019, and it was officially declared a public health emergency of international concern on January 30, 2020. The COVID-19 outbreak and the safety measures taken to control it caused many psychological issues in populations worldwide, such as depression, anxiety, and stress.

**Objective:**

The objectives of this study were to assess the psychological effects of the lockdown due to the COVID-19 outbreak on university students in the United Arab Emirates (UAE) and to investigate the students’ awareness of mobile mental health care apps as well as their attitudes toward the use of these apps.

**Methods:**

A two-part self-administered web-based questionnaire was delivered to students at United Arab Emirates University. The first part of the questionnaire assessed the mental state of the participants using the 12-item General Health Questionnaire (GHQ-12), while the second part contained questions investigating the participants’ awareness of and attitudes toward mental health care apps. Students were invited to fill out the web-based questionnaire via social media and mailing lists.

**Results:**

A total of 154 students participated in the survey, and the majority were female. The results of the GHQ-12 analysis showed that the students were experiencing psychological issues related to depression and anxiety as well as social dysfunction. The results also revealed a lack of awareness of mental health care apps and uncertainty regarding the use of such apps. Approximately one-third of the participants (44/154, 28.6%) suggested preferred functionalities and characteristics of mobile mental health care apps, such as affordable price, simple design, ease of use, web-based therapy, communication with others experiencing the same issues, and tracking of mental status.

**Conclusions:**

Like many groups of people worldwide, university students in the UAE were psychologically affected by the lockdown due to the COVID-19 outbreak. Although apps can be useful tools for mental health care delivery, especially in circumstances such as those produced by the outbreak, the students in this study showed a lack of awareness of these apps and mixed attitudes toward them. Improving the digital health literacy of university students in the UAE by increasing their awareness of mental health care apps and the treatment methods and benefits of the apps, as well as involving students in the app creation process, may encourage students to use these tools for mental health care.

## Introduction

On December 31, 2019, the World Health Organization (WHO) was informed of several cases of pneumonia of unknown cause detected in Wuhan City, China [1]. On January 7, 2020, the cause was reported to be a new coronavirus, which was then referred to as “2019-nCoV” [1]. By January 20, 2020, the virus had spread, and multiple cases were reported in four countries [1]. On January 30, 2020, the WHO declared the outbreak to be a public health emergency of international concern [2]. By February 11, 2020, the virus had spread to more than 24 countries in addition to China, and the coronavirus disease was officially named COVID-19 [3]. By May 2020, the virus had spread to all regions of the world [4]. More than 3 million cases and 248,847 deaths were reported worldwide as of May 4, 2020. To limit the spread and risk of the virus, the WHO advised people to practice social distancing and to stay at home [5]. Countries took different safety measures and precautions to prevent the spread of the disease. Several countries declared obligatory lockdowns, travel was halted and airports were shut down, and many work spaces, schools and universities were closed.

Lockdown-related stressors such as concern about the duration of lockdown, fear of infection, boredom, inadequate information [6], and fear of the unknown [7] had significant psychological effects on people, including posttraumatic stress disorder symptoms, anger, confusion, fear, worry, sadness, and elevated anxiety and stress [6,8]. As part of its application of precautions and safety measures to address the outbreak, the United Arab Emirates (UAE) closed its universities and stopped all related activities. To investigate the impact of the lockdown on university students in the UAE, we conducted a mental health assessment based on the 12-item General Health Questionnaire (GHQ-12). The GHQ-12 has been used in previous studies to assess the mental health of students and has shown positive reliability results. A study using the GHQ-12 to assess the psychological state of Malaysian college students affirmed that the GHQ-12 is a good tool for assessing the overall psychological well-being of students [9]. In another study, the questionnaire was used with Australian college students; the results suggested that the GHQ-12 is a viable tool with good clinical utility and that it can be implemented as part of routine school screening procedures to help identify young people at risk of depressive and anxiety disorders [10]. Another study used the GHQ-12 with Tehrani university students, and it was concluded that the questionnaire is suitable for screening psychopathology in university students [11].

Moreover, the validity of the psychometric properties of the GHQ-12 was asserted in several studies. The GHQ-12 was assessed against the Clinical Interview Schedule (CIS) in three primary care settings in Brazil and was found to be acceptable and valid [12]. The GHQ-12 was also assessed in a sample of dermatological patients against the Skindex-29, which is a tool to evaluate the impact of skin conditions on the quality of life of patients. The results showed that the GHQ-12 is a reliable and valid instrument [13]. Furthermore, the GHQ-12 was reported to be robust and suitable for use as a screening instrument in a study conducted by the WHO in which the 28-item General Health Questionnaire (GHQ-28) was compared to the GHQ-12 [14].

Identifying existing psychological issues is an important step; however, it is more important to deliver mental health care when it is needed. This delivery is currently challenging because of the safety measures being applied to prevent the spread of COVID-19. Telehealth is a health care delivery method that, like tele-education and telework, currently seems to be the safest approach. Telehealth can be applied to mental health care via several methods, one of which is mobile apps. Apps are highly suitable to provide services while safety measures are being applied during the pandemic. They can be used to provide mental health care without need for human contact, and the user can benefit from care delivered via the app without needing to leave their home and risk exposure to the virus. Apps can also help overcome several pre-existing barriers to mental health care delivery in addition to those created by the COVID-19 outbreak, such as cost problems, stigma, and distance or shortage of mental health professionals. Apps have also shown promising results in the management of many mental issues, such as anxiety, depression, and stress [15,16].

This study had two main goals. The first goal was to assess the mental state of university students during the first period of the lockdown caused by the COVID-19 outbreak. The second goal of the study was to investigate the students’ awareness of mobile apps for mental health care and their willingness to use them as well as to discover the features they would like a mental health care app to have and the factors that would encourage them to use such apps. A two-part web-based questionnaire was delivered to United Arab Emirates University (UAEU) students via social media and email during the first two weeks of the lockdown imposed in the UAE. The first part of the form consisted of the GHQ-12 questionnaire, and the second part of the form included questions investigating the students’ attitudes toward mobile apps for mental health care. The study verified the following hypotheses: (h1) the lockdown has an impact on the psychological state of the university students in the UAE; (h2) university students are aware of the existence of mobile apps for mental health care; and (h3) university students have positive attitudes toward mobile mental health care apps and are open to the use of such solutions.

## Methods

### Research Design

This study constitutes two main parts. The objective of the first part was to assess the psychological health of university students. The objective of the second part was to investigate the students’ awareness of and attitudes toward mHealth apps for mental health care.

### Recruitment and Data Collection

A self-administered web-based questionnaire was created using Google Forms and sent to UAEU students via social media and mailing lists. Recruiting participants through social media has been found to be effective and time-efficient [17]. The included participants were a self-selecting sample, as participation in the survey was voluntary and participants were not offered any incentives. The questionnaire consisted of 20 questions and was made available on the internet for two weeks, from March 15, 2020, to March 29, 2020. The questions included 13 predefined multiple-choice questions, 4 yes/no questions, and 3 open questions. None of the questions were personal questions that could reveal the participants’ identity. The questionnaire was tested by the authors before it was sent to students. The estimated time to complete the questionnaire was 4 minutes, which was stated in the questionnaire. The questionnaire also stated that the responses would be anonymous. Prior to data collection, permission was obtained from the relevant authorities at UAEU, and a psychologist was consulted to determine the appropriateness of the questionnaire for the target respondents. The information provided about the questionnaire was based on the Checklist for Reporting Results of Internet E-Surveys (CHERRIES) [18].

### Survey Questions

The web-based questionnaire contained 20 questions. The first three questions aimed to obtain basic information and characteristics of the participants (age, gender, and academic major). The remaining questions were divided into two parts.

#### Part 1: GHQ-12

In this part, we used the GHQ-12 [19] to measure the psychological health of the survey participants. The GHQ-12 can be analyzed as a single dimension psychological health test [20]. However, many researchers have suggested that it can be divided into two or three specific and meaningful factors, in which each factor is composed of several items from the questionnaire. Gribbin and Worsley [21] proposed a three-factor approach: anxiety/depression, social dysfunction, and loss of confidence. Andrich and van Schoubroeck [22] suggested that positively worded items or questions constitute one factor and negatively worded ones constitute another. Politi et al [23] identified two factors: general dysphoria and social dysfunction. Martin [24] proposed three factors: self-esteem, stress, and successful coping. When compared to other methods and applied to different samples, the three-factor model proposed by Gribbin and Worsley [21], including anxiety and depression (4 items), social dysfunction (6 items), and loss of confidence (2 items), was found to give the best fit [25,26]. Therefore, we used this model in this study.

Table 1 presents the 12 items of the GHQ-12. Table 2 presents the association between the GHQ-12 items and the three psychological factors. A study by Gao et al [27] showed that the loadings of the items on their associated factors are very close, ranging from 0.72 to 0.9. The correlation between the three factors was also found to be very high, ranging from 0.83 to 0.9 [27]. Based on these results, and to simplify the assessment, we assumed that all items had the same loadings on their associated factors.

The potential answers to the GHQ-12 items range from “better/healthier than normal” through “same as usual” and “worse/more than usual” to “much worse/more than usual.” The answers reflect the difference between the psychological health states of the participants when they responded to the questionnaire and when they considered their health states to be normal. The answers can be scored in four different ways: GHQ scoring (0-0-1-1), C-GHQ scoring ((0-1-1-1) for negative items and (0-0-1-1) for positive items), Likert scoring (0-1-2-3), and modified Likert scoring (0-0-1-2).

This study investigates the severity of the identified psychological issues in addition to their prevalence; therefore, we used the Likert scoring method to score the answers because it produces a wider and smoother score distribution, which helps to assess severity [28]. Each item of the GHQ-12 had 4 possible answers that were scored from 0 to 3. A higher score indicated a more severe condition. The scores for each answer of the 12 items are presented in Table 1.

**Table 1:** Questions, answers, and scores of the 12-item General Health Questionnaire.

Item ID	Question	Answers	Score
1	Have you recently been able to concentrate on what you are doing?	Better than usual	0
		Same as usual	1
		Less than usual	2
		Much less than usual	3
2	Have you recently lost much sleep over worry?	Not at all	0
		No more than usual	1
		Rather more than usual	2
		Much more than usual	3
3	Have you recently felt you were playing a useful part in things?	More so than usual	0
		Same as usual	1
		Less than usual	2
		Much less than usual	3
4	Have you recently felt capable of making decisions about things?	More so than usual	0
		Same as usual	1
		Less than usual	2
		Much less than usual	3
5	Have you recently felt constantly under strain?	Not at all	0
		No more than usual	1
		Rather more than usual	2
		Much more than usual	3
6	Have you recently felt you couldn’t overcome your difficulties?	Not at all	0
		No more than usual	1
		Rather more than usual	2
		Much more than usual	3
7	Have you recently been able to enjoy your normal day-to-day activities?	More so than usual	0
		Same as usual	1
		Less so than usual	2
		Much less than usual	3
8	Have you recently been able to face up to your problems?	More so than usual	0
		Same as usual	1
		Less so than usual	2
		Much less than usual	3
9	Have you recently been feeling unhappy and depressed?	Not at all	0
		No more than usual	1
		Rather more than usual	2
		Much more than usual	3
10	Have you recently been losing confidence in yourself?	Not at all	0
		No more than usual	1
		Rather more than usual	2
		Much more than usual	3
11	Have you recently been thinking of yourself as a worthless person?	Not at all	0
		No more than usual	1
		Rather more than usual	2
		Much more than usual	3
12	Have you recently been feeling reasonably happy, all things considered?	More so than usual	0
		About same as usual	1
		Less so than usual	2
		Much less than usual	3

**Table 2:** Associations between items and psychological factors of the 12-item General Health Questionnaire.

Psychological factor	Associated item IDs
Anxiety and depression	2, 5, 6, and 9
Social dysfunction	1, 3, 4, 7, 8, and 12
Loss of confidence	10 and 11

The answers of the participants were collected and scored. Based on the maximum score of each psychological factor and the total score, the answers were categorized to represent three severity categories: normal, high, and severe.

For the total score of the GHQ-12, the normal state score category ranged from 0 to 12, the high-risk score category ranged from 13 to 24, and the severe case score category ranged from 25 to 36. For the anxiety and depression factor, the normal state scores ranged from 0 to 4, the high-risk category scores ranged from 5 to 8, and the severe case scores ranged from 9 to 12. For the social dysfunction factor, the normal state scores ranged from 0 to 6, the high-risk scores ranged from 7 to 12, and the severe case scores ranged from 13 to 18. For the “loss of confidence” factor, the normal state scores ranged from 0 to 2, the high risk state included scores of 3 and 4, and the severe case state included scores of 5 and 6.

#### Part 2: Awareness of and Attitudes Toward Mobile Mental Health Apps

The second part of the questionnaire served to investigate the university students’ knowledge of mental health apps and their opinions about the use of these apps for mental health care. The questions were formulated based on questions retrieved from related literature investigating similar topics [29-32]. As shown in Table 3, the questions were formulated mainly to determine if the students had any previous knowledge or experience with mental health apps; understand their willingness to consider using a mental health app; and understand the preferences and factors that could encourage them to use apps for mental health care.

Questions Q1 and Q2 were yes/no questions. Questions Q3 and Q4 had possible answers of “Yes,” “No,” or “I don’t know.” If a participant answered “Yes” to Q4, they were asked to provide the reason for their choice in Q4.1, in which the participant could choose from a predefined list of reasons, including cost problems, stigma related to mental problems, distance from mental health care professionals, shortage of mental health care professionals, and lack of knowledge (information) on mental health; they could also choose the “Other” option and express their own reasons. Q5 was an open question. 

**Table 3:** Questions in Part 2 of the questionnaire related to mental health care apps.

Item ID	Question
1	Have you ever heard of mobile mental health apps?
2	Have you ever used a mobile app for your mental well-being?
3	Would you be open to using a mobile app for your mental well-being in the future?
4	Would you prefer using a mobile app over consulting with a mental health care specialist?
4.1	If yes, why?
5	What would you like to see in an app for mental health care?

#### Correlations

The correlations between the age and the total GHQ-12 score as well as between the gender and the total GHQ-12 score were calculated using the Pearson correlation coefficient, which has a value between +1 and –1. A value of +1 indicates a perfect positive correlation between the sets of data, while a value of –1 indicates a perfect negative correlation. A value of 0 indicates no correlation between the sets; the closer the correlation value is to 0, the weaker the correlation is.

### Summary of the Analytical Strategy

For the GHQ-12 items, the participants answered each GHQ-12 item with “better/healthier than normal,” “same as usual,” “worse/more than usual,” or “much worse/more than usual.” The collected answers were converted to numerical scores based on the Likert scoring (0-1-2-3) method. The scores were regrouped to investigate the psychosocial factors presented in Table 2 (anxiety and depression, social dysfunction, and loss of confidence). A total GHQ-12 score was calculated based on the scores of all the items. The total score and scores of the psychological factors were categorized into three categories: normal state, high-risk state, and severe state. The higher the score, the more severe the participant’s condition.

For the questions related to mental health care apps, the participants answered questions Q1 to Q4 using predefined options. The answers to questions Q1 to Q4 were then quantified and analyzed. For the open questions (Q5 and the “Other “option in Q4.1), the participants provided short answers. The answers of these open questions were then categorized based on their meanings and presented in a list.

## Results

### Sample Characteristics

A total of 154 university students participated in the survey; the majority of the participants were female (113, 73.4%). The academic majors of the majority of the respondents fell into the categories of science, mathematics, and technology (89/154, 57.8%). Table 4 summarizes the main characteristics of the participants.

**Table 4:** Characteristics of the study participants (N=154).

Variable		Value
**Gender, n (%)**		
	Female	113 (73.4)
	Male	41 (26.6)
**Age (years)**		
	15-22, n (%)	118 (76.6)
	≥23, n (%)	36 (23.4)
	Mean (SD)	22.45 (5.87)
**Academic major, n (%)**		
	Science, mathematics, and technology	89 (57.8)
	Business	24 (15.6)
	Health and medicine	10 (6.5)
	Social sciences	5 (3.2)
	Public and social services	5 (3.2)
	Other	21 (13.6)

### Part 1 Results

#### Results by Item

**Figure 1:**
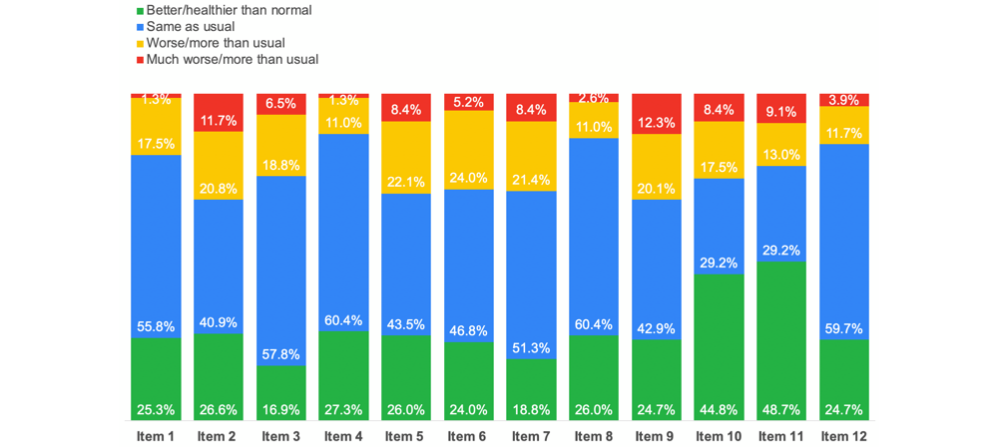
Proportions of participants’ answers to the 12-item General Health Questionnaire.

The results of the analysis of the items identified specific issues that the students suffered from more during the period of the survey. If the participant’s answer to an item indicated a better/healthier state than usual or the same state as usual, the issue investigated by the item was not considered to be more present than usual for the participant; however, if the answer indicated a worse or much worse state than usual, it reflected an abnormal presence of the issue during the survey period. Figure 1 presents the answers to the GHQ-12 items.

One third of the 154 participants (50, 32.5%) had sleep issues (Item 2): 32 (20.8%) reported worse sleep than usual and 18 (11.7%) reported much worse sleep than usual. The same number of participants (50/154, 32.5%) showed elevated states of unhappiness and depression (Item 9), with 31/154 (20.1%) answering worse than usual and 19/154 (12.3%) answering much worse than usual. One-third of the participants (47/154, 30.5%) reported feeling constantly under strain (Item 5), with 34/154 (22.1%) answering worse than usual and 8.4% (13/154) answering much worse than usual. The issues for which participants showed the least elevated states were the capabilities of making decisions and facing problems (Items 4 and 8, respectively); 19/154 participants (12.3%) reported worse capability of making decisions, and 21/154 (13.6%) reported worse ability to face problems.

#### Total Score and Psychological Factors

**Figure 2:**
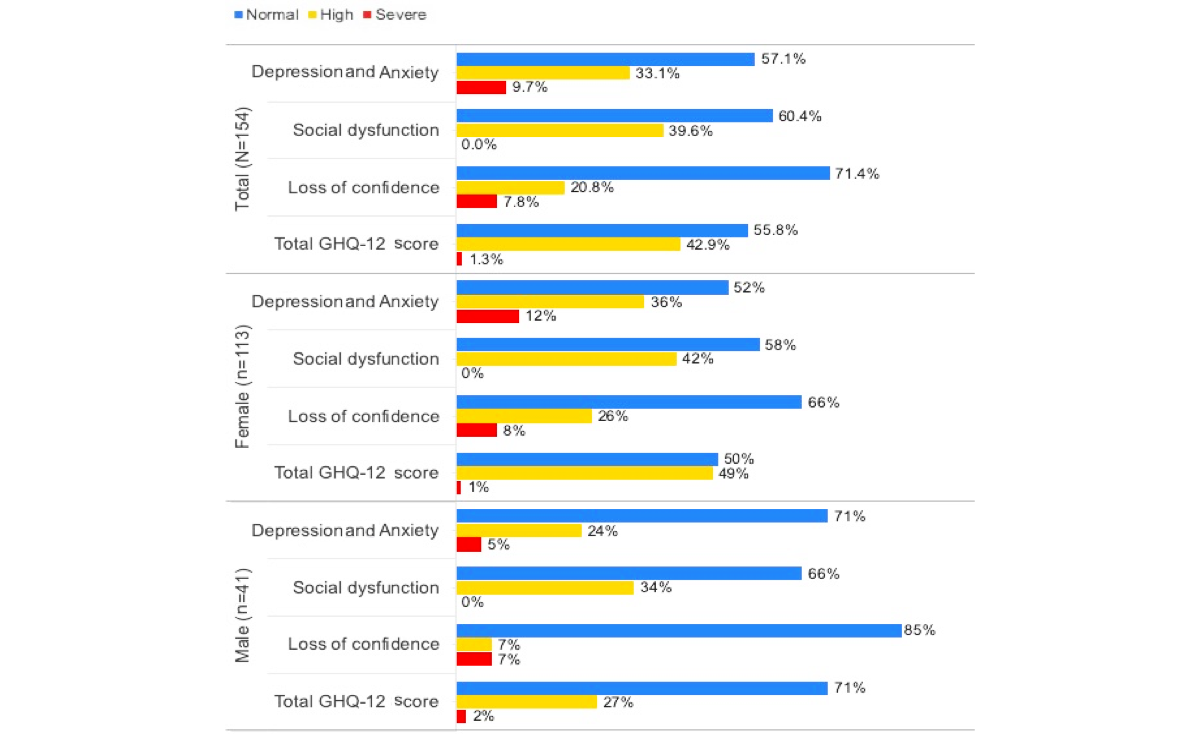
Classification of GHQ-12 answers by severity category and gender. GHQ-12: 12-item General Health Questionnaire.

Analysis of the total scores and the scores of each psychological factor gave a general idea of the students’ mental states and the mental issues they were more susceptible to. Figure 2 presents the severity categories of each psychological factor as well as the total score of the GHQ-12. The figure also presents a classification by gender for each category.

More than one-third of the participants (66/154, 42.9%) had high scores indicating high risk of having mental issues, and a small number of participants (2/154, 1.3%) had very high scores and were classified as severe cases. The issues of the participants mainly seemed to relate to depression and anxiety.

Weak correlations were found between the age of the participants and the total score of the GHQ-12 (–0.101) and between the gender of the participants and the total score of the GHQ-12 (–0.128).

### Part 2 Results

#### Answers to the Questions

**Figure 3:**
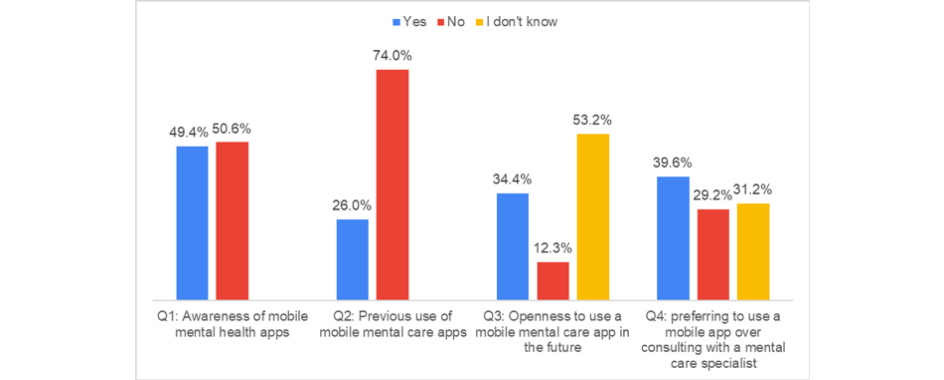
Answers to questions Q1, Q2, Q3, and Q4 pertaining to mental health apps.

Figure 3 presents the responses to questions Q1 to Q4 related to mental health care apps.

**Figure 4:**
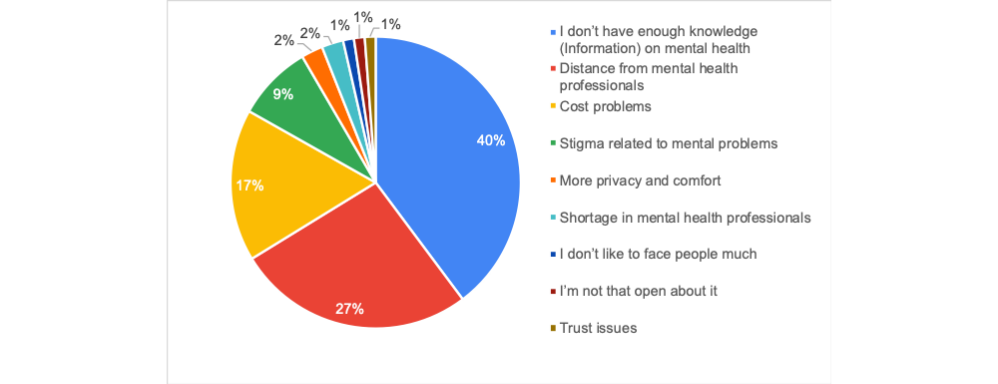
Answers to Q4.1 indicating the participants’ reasons for preferring the use of a mobile app over consulting with a mental health care specialist (n=83).

Half of the participants (78/154, 50.6%) had never heard of mental health care apps (Q1). The majority of the participants (114/154, 74.0%) had never used an app for mental health care (Q2). More than half of the participants (82/154, 53.2%) showed uncertainty regarding their willingness to use mental health care apps in the future (Q3). Of the 154 participants, 83 (53.9%) justified their answers of “Yes” or “I don’t know” to Q4 by the reasons shown in Figure 4.

To identify the features that the students would need or prefer to see in mental health care apps, the participants were asked an open question (Q5) about what would they like to see in an app for mental health care. Almost one-third of the participants (44/154, 28.6%) answered the open question Q5 with the functionality and characteristics that they thought mental health care apps should have. Their answers are grouped and presented in Textbox 1.

**Preferred functionality and characteristics of mental health care apps indicated by the survey participants.**

Functionality

- Web-based therapy
- Communication with others experiencing same issues
- Tracking mental status
- Advice from specialists
- Motivational statements
- Educational content
- Games
- Recommendations of activities and tips
- General health management
- Emergency features
- Tracking of progress
- Stories

Characteristics

- Anonymity
- Simple design and ease of use
- Affordable price
- Diversity of features

### Associations Between the Answers

**Figure 5:**
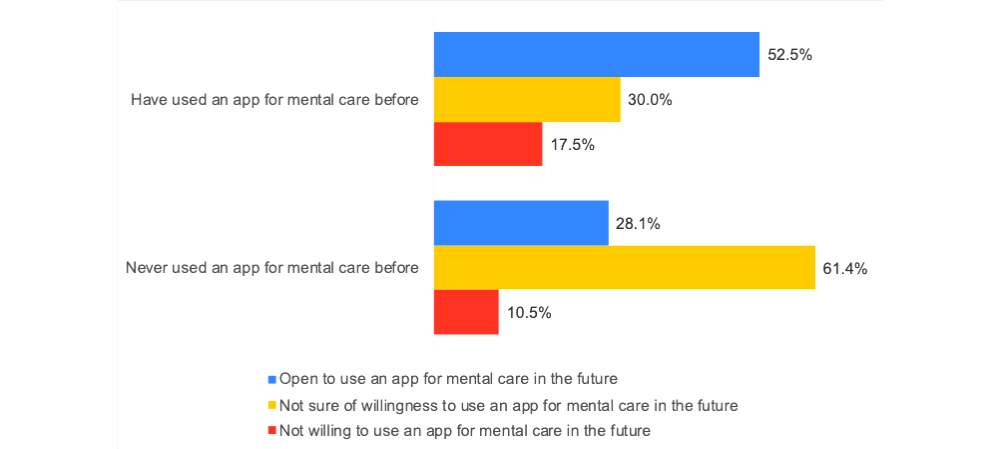
Associations between the answers to Q2 and Q3.

**Figure 6:**
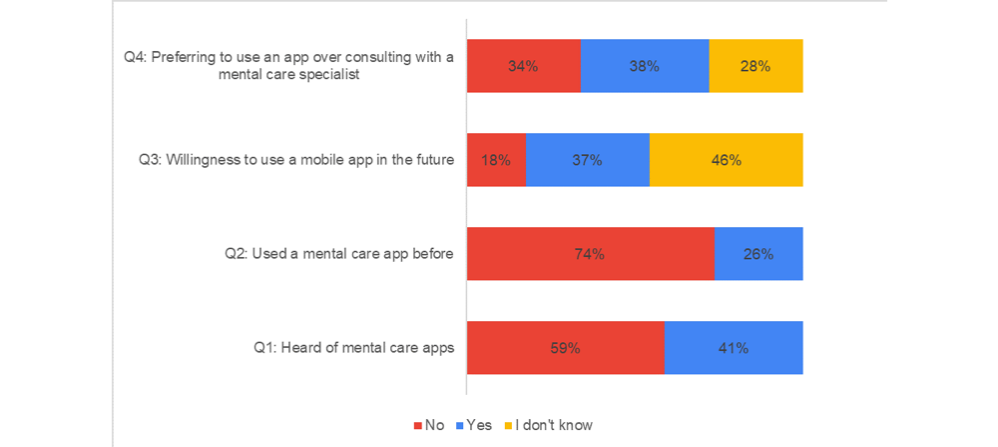
Answers to questions Q1 to Q4 by participants whose total GHQ-12 scores indicated high and severe mental health risk (n=68).

Figure 5 shows the associations between the participants’ previous use of apps for mental health care (Q2) and their willingness to use these apps in the future (Q3). Figure 6 presents the answers to questions Q1 to Q4 by the participants who had high or severe risk scores in Part 1 of the questionnaire.

## Discussion

### Main Findings

The results of Part 1 of our questionnaire showed that more than one-third of the participants (66/154, 42.9%) were experiencing psychological health issues associated with anxiety and depression. The most frequently reported issues related to anxiety and depression were sleep problems, feelings of unhappiness, depression, and being constantly under strain. Given the period of the survey, those results can be linked to the measures taken by universities in the UAE due to the COVID-19 pandemic. The questionnaire was made available on the internet after universities were officially closed to students for two weeks and lectures were being given on the internet. Students were required to adjust to new circumstances and lifestyles and face ambiguity regarding what would happen next concerning their education and future. These findings are consistent with those of other studies conducted in other countries to analyze the psychological impact of the COVID-19 pandemic. Several studies have shown that anxiety, depression, sleep issues [33], and stress [34] are the most common psychological issues caused by the outbreak.

Many students also showed high risk of social dysfunction issues. Items 4 and 8 of the questionnaire were related to the students’ capabilities of making decisions and facing problems, respectively. The nature of these issues implies that they are mainly linked to critical and problematic cases of social dysfunction; also, they may be indicators of serious pre-existing problems that may not necessarily be linked to the COVID-19 lockdown. Social dysfunction issues were mainly related to the lack of enjoyment of daily activities (item 7) and lack of a sense of playing a useful part in things (item 3). This can be linked to the pausing of many activities and the limitations on what students could do at and from home due to the safety measures taken during the outbreak.

We found weak correlations between the age of the participants and the total score of the GHQ-12 (–0.101) and between the gender of the participants and the total score of the GHQ-12 (–0.128). This indicates that university students at any age can be psychologically influenced by the lockdown due to COVID-19. The majority of participants that had high or very high psychological factor scores were female. Women have been reported to be more susceptible to anxiety, fear, and stress [35-37], which may explain this result.

The COVID-19 outbreak has caused many psychological issues among different groups of people, and with the required safety measures, it can be difficult to consult with mental health care professionals even in emergency cases. Alternative remote mental health care solutions are needed during the pandemic, just as distance learning was required as an alternative to in-school learning. Apps are convenient solutions during the current pandemic, as they provide mental health care via mobile devices, which are owned by the majority of people in the UAE, particularly the younger generation [38]. Apps for mental health care can also help overcome some pre-existing mental health care barriers, such as stigma, cost, and distance from mental health care professionals and institutions [39,40]. Apps have also been reported to be effective to alleviate anxiety, stress, and depression [15,16]. The results showed that the majority of participants (78/154, 50.6%), who are university students in the UAE, had never heard of mobile mental health care apps, and 114/154 respondents (74.0%) had never used this type of app. This was also reflected in their attitudes toward these solutions, as 82/154 participants (53.2%) were unsure about their willingness to use an app for mental health care in the future. One-third of the participants (48/154, 31.2%) were not sure if they would prefer using an app to consulting with a mental health care professional. These results were also persistent when analyzing answers of participants who had high or very high general GHQ-12 scores, as shown in Figure 6. These participants may be in need of such apps. The lack of knowledge and use of apps may be due to the fact that the younger generation mainly associates apps with games, communication, or other activities not related to health or mental health in particular [41]. A person who is not familiar with the concept of a mental health care app may not think to search for one.

There is a need for digital health literacy, particularly for mental health care, in the UAE. This can be provided using social networks such as Instagram and Twitter or via widely used websites such as YouTube, as these platforms reach a large number of people and can help spread knowledge about available mental health care apps.

Some participants (12/114, 10.5%) who had never used such apps expressed unwillingness to use one in the future; this may be due to a lack of knowledge or trust regarding these apps. Involving mental health professionals in the design of apps for mental health could give the apps more credibility and encourage university students to be more open toward using them. It is noteworthy that a group of participants who had used apps for mental health care before (7/40, 17.5%) expressed unwillingness to use them again in the future; this may be due to poor usability and/or lack of functionality of the used apps. End users should be involved in the creation of mHealth apps [42] to improve the adoption of these solutions. Integrating gamification features in these apps may also be a solution to make the apps more enjoyable and engaging and to encourage users to continue using them.

The majority of participants (109/154, 70.8%) answered “Yes” or “I don’t know” when asked if they preferred using an app to consulting with a mental health care professional; these participants justified their choices as being mainly due to their lack of knowledge of mental health. Apps can be a useful means for people to educate themselves about mental issues and to obtain easy access to information. The participants also suggested that educational content could be provided in mental health care apps. It must be noted that mental health care apps are not a substitute for professional care. These apps can aid the management of certain mental issues and deliver certain treatment methods; however, seeking professional help is imperative to treat serious mental health issues.

Stigma was among the expressed reasons for preferring mental health care apps. Apps can provide anonymity and confidentiality when only users can access their content, which helps overcome stigma barriers. The aforementioned listed reasons were consistent with the students’ preferred functionality and characteristics, such as web-based therapy, advice from specialists, anonymity to help overcome stigma, and affordable price to overcome cost issues. The results of this study are consistent with the results of previous studies reporting that stigma, cost, and distance from mental health care professionals are the main barriers to mental health care delivery [39,40]. Simple design and ease of use were among the characteristics suggested by the participants. They felt that the apps should be easy to use, easy to learn, and provide enjoyable user experience. The aforementioned functionality and characteristics suggested by the students could be established as requirements of mental health apps for university students in the UAE.

Although mental health care apps are a convenient means to overcome many mental health care barriers, it must be noted that for an app to be beneficial, it must ensure safety, privacy, security, and confidentiality of users’ data. Available apps for mental health care differ in their quality, effectiveness, and security measures. Psychological health is a sensitive subject; therefore, the user should check the permissions required by these apps and the treatment approaches they provide before using them.

### Limitations

This study may have some limitations. First, the survey was conducted during the early stages of lockdown in the UAE; the psychological state of the students may have changed since then, and they are likely to be experiencing more psychological issues. Second, the difference in the number of participants by gender may have affected the psychological investigation results; the majority of participants were female, and women have been reported to more susceptible to certain psychological issues. It should be noted that female students represent 81% of the entire UAEU student population [43], which may also have impacted the results. Third, comparing the results with a prelockdown investigation of the psychological state of university students in the UAE using the GHQ-12 would have improved the discussion of the results. However, to the best of our knowledge, no such investigation exists. Fourth, a broader number of participants may have been included if the survey had not been conducted during a pandemic. Fifth, conducting semistructured interviews could have improved the discussion of the results. However, as the study was conducted at the beginning of the lockdown due to the COVID-19 pandemic, delivering the questions through a web-based form was our best and only choice. Finally, given that more than half of the respondents were unfamiliar with mental health apps, the conclusions regarding the benefits of the use of these apps were not based on the results of the questionnaire but on results of previous studies investigating the effectiveness and advantages of mental health care apps.

### Conclusions and Future Work

The COVID-19 outbreak and the applied safety measures to limit its spread have caused many psychological issues worldwide. Our psychological assessment test of university students based on the GHQ-12 showed that more than one-third of the participants were experiencing issues related to depression and anxiety as well as to social dysfunction, which confirmed our first hypothesis. In contrast, our second hypothesis was found to be unsupported, as the majority of students showed a lack of awareness of mental health care apps. The students also showed mixed attitudes and uncertain willingness to use such apps, which does not support our third hypothesis. The participants proposed characteristics and functionalities of mental health care apps that could encourage them to use such apps. We encourage developers of mental health apps to consider these suggestions, especially when targeting university students or the younger generation. We do believe that the findings of this study may assist researchers and practitioners investigating the impact of the COVID-19 outbreak on the psychological health of university students.

In future work, we intend to build on the results of this study and develop a mobile app to help university students cope with mental health issues.

## Abbreviations

CHERRIES: Checklist for Reporting Results of Internet E-Surveys
GHQ-12: 12-item General Health Questionnaire
GHQ-28: 28-item General Health Questionnaire
mHealth: mobile health
UAE: United Arab Emirates
UAEU: United Arab Emirates University
WHO: World Health Organization

## References

[1] (2020). Novel Coronavirus (2019-nCoV) Situation Report - 1. World Health Organization.

[2] (2020). Novel Coronavirus (2019-nCoV) Situation Report - 11. World Health Organization.

[3] (2020). Novel Coronavirus (2019-nCoV) Situation Report – 22. World Health Organization.

[4] (2020). Coronavirus disease (COVID-19) Situation Report – 104. World Health Organization.

[5] Coronavirus disease (COVID-19) advice for the public. World Health Organization.

[6] Brooks S, Webster R, Smith L, Woodland L, Wessely S, Greenberg N, Rubin G (2020). The psychological impact of quarantine and how to reduce it: rapid review of the evidence. Lancet.

[7] Shihabuddin L (2020). How to Manage Stress and Anxiety from Coronavirus (COVID-19). RWJBarnabas Health.

[8] Murray J, Sherwood H (2020). Anxiety on rise due to coronavirus, say mental health charities. The Guardian.

[9] Zulkefly SN, Baharudin R (2010). Using the 12-item General Health Questionnaire (GHQ-12) to Assess the Psychological Health of Malaysian College Students. GJHS.

[10] Baksheev GN, Robinson J, Cosgrave EM, Baker K, Yung AR (2011). Validity of the 12-item General Health Questionnaire (GHQ-12) in detecting depressive and anxiety disorders among high school students. Psychiatry Res.

[11] Yaghubi H (2012). Validity and factor structure of the General Health Questionnaire (GHQ-12) in university students. IJBS.

[12] Mari JJ, Williams P (1985). A comparison of the validity of two psychiatric screening questionnaires (GHQ-12 and SRQ-20) in Brazil, using Relative Operating Characteristic (ROC) analysis. Psychol Med.

[13] Picardi A, Abeni D, Pasquini P (2001). Assessing psychological distress in patients with skin diseases: reliability, validity and factor structure of the GHQ-12. J Eur Acad Dermatol Venereol.

[14] Goldberg DP, Gater R, Sartorius N, Ustun TB, Piccinelli M, Gureje O, Rutter C (1997). The validity of two versions of the GHQ in the WHO study of mental illness in general health care. Psychol Med.

[15] Lipschitz J, Miller CJ, Hogan TP, Burdick KE, Lippin-Foster R, Simon SR, Burgess J (2019). Adoption of Mobile Apps for Depression and Anxiety: Cross-Sectional Survey Study on Patient Interest and Barriers to Engagement. JMIR Ment Health.

[16] Coulon SM, Monroe CM, West DS (2016). A Systematic, Multi-domain Review of Mobile Smartphone Apps for Evidence-Based Stress Management. Am J Prev Med.

[17] Gaupp-Berghausen M, Raser E, Anaya-Boig E, Avila-Palencia I, de Nazelle A, Dons E, Franzen H, Gerike R, Götschi T, Iacorossi F, Hössinger R, Nieuwenhuijsen M, Rojas-Rueda D, Sanchez J, Smeds E, Deforth M, Standaert A, Stigell E, Cole-Hunter T, Int Panis L (2019). Evaluation of Different Recruitment Methods: Longitudinal, Web-Based, Pan-European Physical Activity Through Sustainable Transport Approaches (PASTA) Project. J Med Internet Res.

[18] Eysenbach G (2004). Improving the quality of Web surveys: the Checklist for Reporting Results of Internet E-Surveys (CHERRIES). J Med Internet Res.

[19] Goldberg DP (1972). The detection of psychiatric illness by questionnaire: A technique for the identification and assessment of non-psychotic psychiatric illness.

[20] Buck N, Gershuny J, Rose D, Scott J (1994). Changing Households: The British Household Panel Survey 1990 -1992.

[21] Worsley A, Gribbin CC (1977). A factor analytic study on the twelve item general health questionnaire. Aust N Z J Psychiatry.

[22] Andrich D, van Schoubroeck L (1989). The General Health Questionnaire: a psychometric analysis using latent trait theory. Psychol Med.

[23] Politi PL, Piccinelli M, Wilkinson G (1994). Reliability, validity and factor structure of the 12-item General Health Questionnaire among young males in Italy. Acta Psychiatr Scand.

[24] Martin AJ (1999). Assessing the multidimensionality of the 12-item General Health Questionnaire. Psychol Rep.

[25] Mäkikangas A, Feldt T, Kinnunen U, Tolvanen A, Kinnunen M, Pulkkinen L (2006). The factor structure and factorial invariance of the 12-item General Health Questionnaire (GHQ-12) across time: evidence from two community-based samples. Psychol Assess.

[26] Cheung YB (2002). A confirmatory factor analysis of the 12-item General Health Questionnaire among older people. Int J Geriatr Psychiatry.

[27] Gao F, Luo N, Thumboo J, Fones C, Li S, Cheung Y (2004). Does the 12-item General Health Questionnaire contain multiple factors and do we need them?. Health Qual Life Outcomes.

[28] (2020). General Health Questionnaire. GL Assessment.

[29] Sreejith G, Menon V (2019). Mobile Phones as a Medium of Mental Health Care Service Delivery: Perspectives and Barriers among Patients with Severe Mental Illness. Indian J Psychol Med.

[30] Sukmawati I, Ardi Z, Ifdil I, Zikra Z (2019). Development and Validation of Acceptability of Mental-Health Mobile App Survey (AMMS) for Android-based Online Counseling Service Assessment. J Phys Conf Ser.

[31] Miller KE, Kuhn E, Yu J, Owen JE, Jaworski BK, Taylor K, Blonigen DM, Possemato K (2019). Use and perceptions of mobile apps for patients among VA primary care mental and behavioral health providers. Prof Psychol Res Pr.

[32] Atallah N, Khalifa M, El Metwally A, Househ M (2018). The prevalence and usage of mobile health applications among mental health patients in Saudi Arabia. Comput Methods Programs Biomed.

[33] Huang Y, Zhao N (2020). Generalized anxiety disorder, depressive symptoms and sleep quality during COVID-19 outbreak in China: a web-based cross-sectional survey. Psychiatry Res.

[34] Wang C, Pan R, Wan X, Tan Y, Xu L, Ho CS, Ho RC (2020). Immediate Psychological Responses and Associated Factors during the Initial Stage of the 2019 Coronavirus Disease (COVID-19) Epidemic among the General Population in China. Int J Environ Res Public Health.

[35] Bhui K, Fletcher A (2000). Common mood and anxiety states: gender differences in the protective effect of physical activity. Soc Psychiatry Psychiatr Epidemiol.

[36] Keogh E, Hamid R, Hamid S, Ellery D (2004). Investigating the effect of anxiety sensitivity, gender and negative interpretative bias on the perception of chest pain. Pain.

[37] Lewinsohn PM, Gotlib IH, Lewinsohn M, Seeley JR, Allen NB (1998). Gender differences in anxiety disorders and anxiety symptoms in adolescents. J Abnorm Psychol.

[38] (2019). Smartphone penetration rate in the MENA 2018, by selected country. Statista.

[39] Lingley-Pottie P, McGrath PJ, Andreou P (2013). Barriers to Mental Health Care. Adv Nurs Sci.

[40] Andrade LH, Alonso J, Mneimneh Z, Wells JE, Al-Hamzawi A, Borges G, Bromet E, Bruffaerts R, de Girolamo G, de Graaf R, Florescu S, Gureje O, Hinkov HR, Hu C, Huang Y, Hwang I, Jin R, Karam EG, Kovess-Masfety V, Levinson D, Matschinger H, O'Neill S, Posada-Villa J, Sagar R, Sampson NA, Sasu C, Stein DJ, Takeshima T, Viana MC, Xavier M, Kessler RC (2013). Barriers to mental health treatment: results from the WHO World Mental Health surveys. Psychol Med.

[41] (2016). Middle East: daily life activities via smartphone 2016 Published by Statista Research Department, Jun 10, 2016 This statistic shows the frequency of life activities internet-going smartphone users performed daily in the Middle East, in 2016, by select country. During the survey period it was found that 73 percent of internet-going smartphone users in the UAE took photos via smartphone daily. Daily life activities performed on smartphones in the Middle East, as of 2016, by select country. Statista.

[42] Ouhbi S, Karampela M, Isomursu M (2019). Integrating Users Logic Into Requirements Engineering for Connected Healthcare co-Design. Proceedings of the 14th International Conference on Evaluation of Novel Approaches to Software Engineering - Volume 1: ENASE.

[43] United Arab Emirates University.

